# Ecological Structure of Recent and Last Glacial Mammalian Faunas in Northern Eurasia: The Case of Altai-Sayan Refugium

**DOI:** 10.1371/journal.pone.0085056

**Published:** 2014-01-13

**Authors:** Věra Pavelková Řičánková, Jan Robovský, Jan Riegert

**Affiliations:** Department of Zoology, Faculty of Science, University of South Bohemia, České Budějovice, Czech Republic; University of York, United Kingdom

## Abstract

Pleistocene mammalian communities display unique features which differ from present-day faunas. The paleocommunities were characterized by the extraordinarily large body size of herbivores and predators and by their unique structure consisting of species now inhabiting geographically and ecologically distinct natural zones. These features were probably the result of the unique environmental conditions of ice age ecosystems. To analyze the ecological structure of Last Glacial and Recent mammal communities we classified the species into biome and trophic-size categories, using Principal Component analysis. We found a marked similarity in ecological structure between Recent eastern Altai-Sayan mammalian assemblages and comparable Pleistocene faunas. The composition of Last Glacial and Recent eastern Altai-Sayan assemblages were characterized by the occurrence of large herbivore and predator species associated with steppe, desert and alpine biomes. These three modern biomes harbor most of the surviving Pleistocene mammals. None of the analyzed Palearctic Last Glacial faunas showed affinity to the temperate forest, taiga, or tundra biome. The Eastern part of the Altai-Sayan region could be considered a refugium of the Last Glacial-like mammalian assemblages. Glacial fauna seems to persist up to present in those areas where the forest belt does not separate alpine vegetation from the steppes and deserts.

## Introduction

The unique structure of Pleistocene mammalian communities has drawn the attention of scientists for many decades [1]–[7]. The extraordinary large body size of some Pleistocene mammals (e.g., mammoths, giant deer, or cave bear) and composition of the ice age communities have no analogies in the present-day faunas [8]–[9]. The Pleistocene communities consisted of species which now inhabit geographically and ecologically distinct natural zones (tundra, forest, steppe, savanna) [5], [8]. Arctic reindeer and musk-ox thus lived in sympatry with e.g. steppe horse and bison and/or with forest elk and roe deer [4], [10].

The reason for such a peculiar structure of Last Glacial (corresponding with Weichselian Glaciation) mammalian assemblages is probably associable with the unique environmental conditions of ice age ecosystems [8], [10], [11]. The non-taxonomic, ecological structure of a mammalian community (∼ its guild composition according to e.g. Simberloff & Dayan [12]) is determined mainly by environmental factors such as climate, type of biome or vegetation heterogeneity [13]–[15]. Each type of ecosystem (e.g. woodland or arid ones) is characterized by a specific trophic-size structure of its mammalian community [5], [13], [16]. Mammalian communities from areas with similar regional climates tend to converge to similar community structures [15]. Historical factors also play an important role in forming community structure, but they operate on a different, probably much longer timescale than environmental factors [14]–[15].

The non-analogue Last Glacial communities evolved in a cold and dry continental climate, which supported highly heterogeneous vegetation and landscape structure, usually described as tundra-steppe or mammoth steppe [4], [8], [10], [17].

The Pleistocene tundra-steppe ecosystem was quite heterogeneous locally but displayed a relatively high degree of homogeneity on the continental scale. This ecosystem covered wide areas of the northern part of the globe, thrived for approximately 100,000 years without major changes, and then suddenly went extinct about 12,000 years ago [4], [10].

Surprisingly, environmental conditions similar to the Last Glacial period have been found in the Central Eurasian Altai-Sayan mountains [18]. This climatic analogue has recently been supported by biological data. Recent findings of the paleo-biome reconstruction [19]–[20] and pollen-analytical research [21]–[23] suggest that present-day Altai-Sayan landscapes could be considered the closest modern analogy to the Last Glacial environments. The area is currently inhabited by mollusc assemblages that were characteristic of full-glacial environments across large areas in Eurasia but went extinct in the regions that experienced considerable climatic change, namely in Europe [24]. Simulated paleovegetation maps based on paleoclimatic models and plant functional types have also suggested considerable stability in Central Eurasia over the last 40,000 years [25], [26]. Detailed analysis of the Altai Late Pleistocene assemblages of small mammals revealed that no significant changes occurred between the cold phase of the Pleistocene and the Holocene [27]. The environment of this region can thus be considered as conservative and stable.

In this study, we compared the ecological structure of Recent Altai-Sayan mammalian assemblages to the ecological structure of Last Glacial fauna of Altai-Sayan and several adjacent regions as well as to Recent mammalian communities from various natural zones of northern Eurasia. In order to examine the most important structural characteristics of Pleistocene assemblages, we assigned individual species according to their biome and the trophic-size categories. Given the analysis of Willis et al. [28] and Rodrigues et al.[7], [29] showing that glacial vegetation consisted of steppe, tundra and forest and mammalian fauna was characterized by large herbivores and predators, we would expect that glacial communities are characterized (and differ from Recent communities) by the co-occurrence of steppe, tundra, and forest species [28], and by high a proportion of large herbivores and predators [29]. We hypothesize that, given the environmental stability of central Eurasia [18], Altai-Sayan Recent assemblages will be more similar to the glacial communities than to any other Recent community [22]–[23].

## Materials and Methods

### Regions and localities

**Figure 1:**
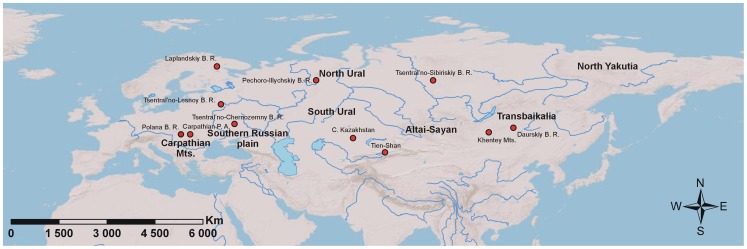
Location map of Recent and Pleistocene Palearctic localities used in the analyses. Abbreviations: B. R. - Biosphere Reserve; P. A. - Protected Area; Mts. - mountains

To compare the ecological compositions of Recent and Last Glacial faunas, lists of mammalian species for 14 Recent and seven Last Glacial localities were collected (Fig. 1, see Table S1). The areas were selected in order to cover most of the Palearctic Realm above 35° N, to include well documented Last Glacial localities, and to be compatible with the WWF eco-regions (e.g., Altai-Sayan, Caucasus, Carpathian Mountains). Taking into account (i) the scarcity of paleontological localities, (ii) the general incompleteness of the fossil record, (iii) and relative homogeneity of the glacial fauna, larger regions were used as units for the analysis of Last Glacial faunas. This grouping of data, therefore, helps to average taphonomic biases [29]. The experimental mixture of modern communities tends to increase the taxonomic richness but does not significantly modify the overall ecological diversity [30].

**Figure 2:**
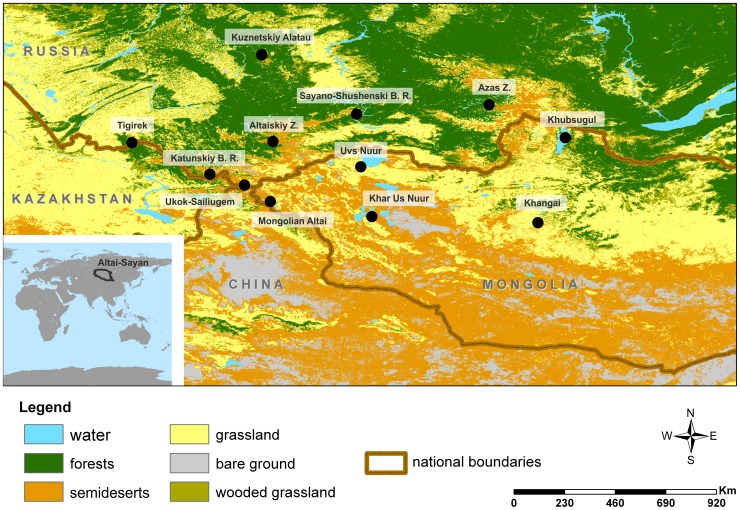
Location map of examined localities in the Altai-Sayan region. Abbreviations: B. R. - Biosphere Reserve; Z. – Zapovednik (National Park). Dashed line: state borders, solid line: Altai-Sayan region borders according to WWF ecoregion.

For the analysis of Recent mammalian faunas we used smaller, ecologically homogeneous areas (see Table S1). The Recent fauna of Altai-Sayan region has been assigned to 12 areas, covering most of the region’s heterogeneity (Fig. 2; Table S2).

### Species

The Recent mammalian communities refer to the interval from now to approximately the 16^th^ century AD in order to respect the IUCN definition of “recent extinction” [31] and to eliminate taxonomical uncertainties. The Last Glacial refers here to the last glacial period of the Late Pleistocene, corresponding with the Weichselian Glaciation. The Last Glacial communities were dated from approximately 125,000 to 12,000 BP, i.e. they included the time interval from MIS 5d to MIS 2 [32] which is well defined in the geological/fossil record [33], [34]. The early-Holocene fauna was not included in our analyses.

Presence/absence of 379 mammalian species in Recent and Last Glacial regions were recorded.. The Recent species include extant (or extinct in the historical period) autochthonous elements but not allochthonous elements associated with human unintended or intentional activities (see Notes S1). The marine mammals (Cetacea, Pinnipedia) and island endemics (though geographically associated with the analyzed regions) were excluded from analyses. Bats (Chiroptera) have been removed from the list of examined species for trophic-size categories, following Rodríguez [7]. Domesticated mammals were not considered.

Some Recent species are not diagnosable in the fossil record owing to the lack of diagnostic morphological, cytogenetic, and/or molecular characters. We therefore fused some closely related mammalian species into single operational taxonomic units (for details see Notes S1). No permits were required for the described study, which complied with all relevant regulations.

### Biome and trophic-size structure of the faunas

The ecological structure of a community is defined as the number of species in different ecological groups or categories, i.e. species with similar trophic habits, body size and ecological requirements [7]. The species were classified according to two schemes:

(i) The species’ affinity to a particular biome (a group of terrestrial ecosystems with similar climates and vegetation structure): we recognized (1) tundra, (2) taiga, (3) deciduous forest, (4) steppe, (5) alpine grassland, and (6) desert species, following Duff & Lawson [35] and Wilson & Reeder [36].

(ii) Trophic and body-size categories combining information about trophic habits, locomotor abilities, microhabitat, and body size of the species examined cf. [7]): (1) aquatic predator (e.g. Eurasian otter *Lutra lutra*); (2) small terrestrial predator (e.g. red fox *Vulpes vulpes*); (3) large terrestrial predator (e.g. wolf *Canis lupus*); (6) aquatic predator of invertebrates (e.g. Russian desman *Desmana moschata*); (7) subterranean predator of invertebrates (e.g. Siberian mole *Talpa altaica*); (9) small terrestrial predator of invertebrates (e.g. Eurasian shrew *Sorex araneus*); (11) small terrestrial omnivore (e.g. Altai birch mouse *Sicista napaea*); (12) large terrestrial omnivore (e.g. brown bear *Ursus arctos*); (13) arboreal omnivore (e.g. forest dormouse *Dryomys nitedula*); (14) small terrestrial herbivore (e.g. common vole *Microtus arvalis*); (15) small sized foregut fermenter (e.g. goitered gazelle *Gazella subgutturosa*); (16) medium sized foregut fermenter (e.g. reindeer *Rangifer tarandus*); (17) large sized foregut fermenter (e.g. red deer *Cervus elaphus*); (18) small sized hindgut fermenter (e.g. mountain hare *Lepus timidus*); (20) large sized hindgut fermenter (e.g. Asiatic wild ass *Equus hemionus*); (21) subterranean herbivore (e.g. Siberian zokor *Myospalax myospalax*); (22) arboreal herbivore (e.g. Eurasian red squirrel *Sciurus vulgaris*); (23) aquatic herbivore (e.g. European water vole *Arvicola amphibius*).

### Data analyses

To visualize the overall similarity of Recent and Last Glacial areas according to presence/absence of the mammalian species, we used all 21 regions/localities as “samples” and ecological categories as “species” for Principal Component Analysis (PCA). We also performed analyses complementary to PCA using non- metric multidimensional scaling (NMDS) with Bray-Curtis dissimilarity indices (CANOCO for Windows software [37] ). We expressed the proportion of species associated with each biome and trophic-size category as percentages in the input data matrix (see Table S3). The percentages were log-transformed and standardized by species. The sensitivity of PCA was controlled by analyzing datasets after removing rare categories (biomes: tundra and desert, trophic-size: aquatic predator, aquatic predator of invertebrates, subterranean predator of invertebrates, arboreal omnivore, small-sized foregut fermenter, subterranean herbivore).

## Results

### (1) Faunas classified according to species-biome associations

**Figure 3:**
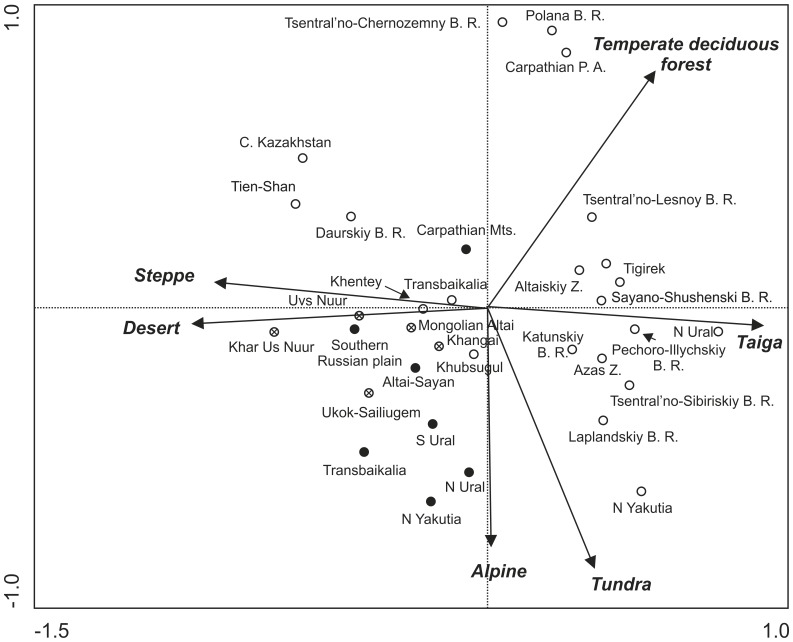
Projection scores of mammalian communities for studied localities (PCA). Species were classified according to their presence in a particular biome. The first two axes describe 89.8% of variance. Open circles – Recent assemblages; crossed circles – Recent eastern Altai assemblages; full circles – Last Glacial assemblages

Composition of Last Glacial faunas was characterized by the co-occurrence of steppe, desert, alpine, and tundra species (Fig. 3). The first axis of the ordination space was determined by the steppe/desert-to-taiga gradient, whereas presence/absence of the alpine faunas was strongly correlated with the second axis. The first two axes explain 89.9% of variance (see Table S4 for detailed results and comparison with NMDS analyses). The Last Glacial faunas were scattered in the area between the steppe/desert and alpine faunas, with no affinities to the tundra and taiga ones. The Last Glacial faunas of North Yakutia and North Ural were characterized by the presence of alpine faunal elements, and did not resemble any of the Recent localities. The faunas of Recent eastern Altai-Sayan localities (with high proportion of grasslands) were characterized by the co-occurrence of steppe, desert, and alpine species (as well as their Last Glacial counterparts), and were more similar to some Last Glacial faunas than to faunas of any other Recent areas. The NMDS analysis and PCA without rare biomes showed similar results to PCA (see Fig. S1 and Fig. S2).

**Figure 4:**
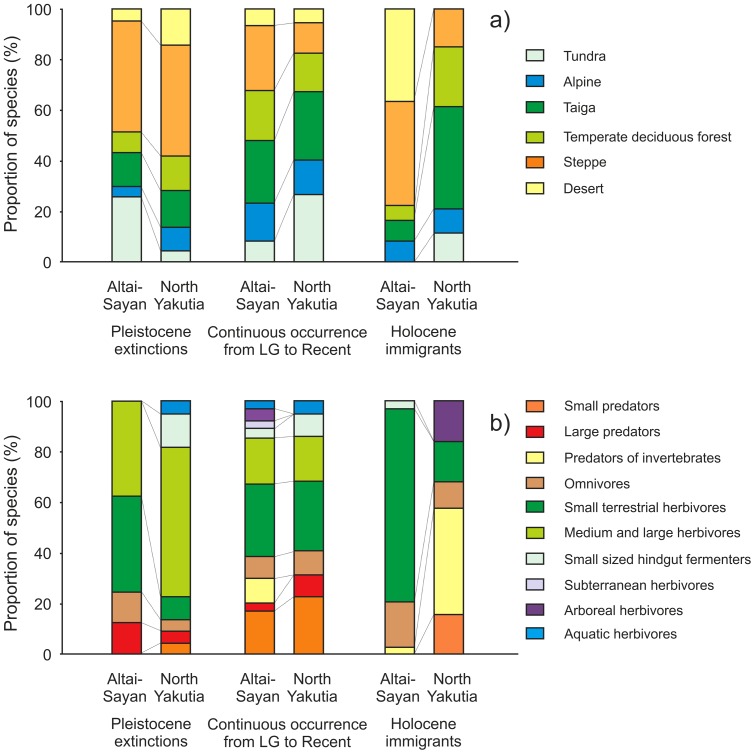
The differences in ecological structure between Altai-Sayan and North Yakutia faunas. Comparison of the proportion of species assigned to biome (a) and trophic-size (b) categories between the regions.

Comparison of the proportion of species assigned to biome categories between Altai-Sayan and North Yakutia regions (Fig. 4a) revealed that the main difference between the regions consisted of a high proportion of forest and taiga species among Holocene immigrant species in North Yakutia and desert species in Altai-Sayan.

### (2) Faunas classified according to trophic-size structure

**Figure 5:**
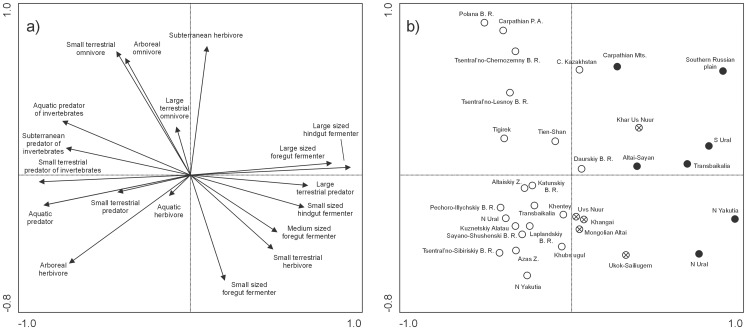
Projection scores of trophic-size community structure (PCA). The first two axes describe 63.4% of variance. Open circles – Recent assemblages; crossed circles – Recent eastern Altai assemblages; full circles – Last Glacial assemblages.

The gradient between Last Glacial and Recent faunas along the first axis (Fig. 5) was determined by the ratio of insectivores and aquatic predators to large ungulates and predators. The Last Glacial faunas were characterized by a high proportion of the large-sized terrestrial predators (e.g., lion, wolf), large-sized foregut fermenters (e.g., steppe bison, deer, camel), and large-sized hindgut fermenters (e.g., mammoth, horse). Recent communities were characterized by the presence of small terrestrial predators of invertebrates, aquatic predators, subterranean predators of invertebrates, and aquatic predators of invertebrates (Fig. 5a). The occurrence of arboreal and small terrestrial omnivores and subterranean herbivores was positively correlated, while the occurrence of small-sized foregut fermenters was correlated negatively to the above mentioned cluster along the second axis (Fig. 5a). The first two axes explain 63.4% of variance. (see Table S4 for detailed results and comparison with NMDS analyses).

All Last Glacial localities were situated on the right side of the diagram (Fig. 5b), together with the Recent faunas from Khar Us Nuur NP and Ukok-Sailiugem localities (both E Altai). The Khar Us Nuur NP is more similar to the Last Glacial Altai-Sayan fauna than to any Recent locality. The Recent fauna of Tigirek (SW Altai) differs from the other Altai-Sayan localities, as it is situated closer to the European temperate-forest localities (Fig. 5b) characterized by a larger proportion of small terrestrial and arboreal omnivores (Fig. 5a). The Last Glacial assemblage of the Carpathian Mountains was more similar to the Recent community of Central Kazakhstan than to any other assemblage. The NMDS analysis and PCA without rare trophic-sizes showed similar results to PCA (see Fig. S3 and Fig. S4).

The main difference between the proportion of species assigned to trophic-size categories in Altai-Sayan and North Yakutia regions (Fig. 4b) consisted of a high proportion of arboreal herbivores and predators of invertebrates among Holocene immigrant species in North Yakutia.

## Discussion

We found a marked similarity in ecological structure between Recent Altai-Sayan mammalian assemblages and Last Glacial paleocommunities. The ecological structure of mammalian communities confirmed the possible persistence of the Last Glacial-like fauna in the present-day Altai-Sayan region, as the Recent assemblages of eastern Altai-Sayan (i.e. Ukok-Sailiugem, Khar Us Nuur, and Uvs Nuur) were more similar to various glacial localities than to the communities of any other Recent areas. Glacial communities have close modern analogues in the three eastern Altai-Sayan areas where e.g. reindeer and saiga antelope still live in sympatry [38].

Our results are congruent with other evidence supporting the persistence of Pleistocene biota in the Altai-Sayan region. Rodríguez [7] reported a similarity between the ecological structure of present-day mammalian communities from Central Eurasia (Uvs Nuur and Great Gobi) and Iberian Pleistocene communities. The Altai Mountains are an important refugium for full-glacial snail faunas, as recently documented by Horsák et al. [24] and Hoffmann et al. [39]. Kuneš et al. [22] and Pelánková & Chytrý [23] demonstrated a close similarity between glacial pollen samples from central Europe and modern surface-pollen spectra from the Altai-Sayan region. Fossil pollen spectra from the Altai and adjacent regions indicate little difference between modern biomes of this region and those reconstructed for the Last Glacial Maximum [20]. Similar conditions possibly occur in climatically stable areas of North American cold deserts. Fossil evidence from the Great Basin indicate that Pleistocene plant assemblages are comparable with the modern ones [40].

According to vegetation studies, three major biomes occurred widely in the Pleistocene: steppe, tundra, and taiga [28], [41]–[42]. In contrast, the composition of the Last Glacial (and Recent eastern Altai-Sayan) mammalian faunas was characterized by the co-occurrence of steppe, desert and alpine species. These three modern biomes harbor most of the surviving Last Glacial mammals. The importance of the desert biome was probably more pronounced in the examined region of southern Russian plain and central Asia in comparison to the northern and western part of Eurasia.

Glacial-like communities still persist in areas where the forest belt does not separate alpine vegetation from the steppes and (semi)deserts. High aridity in eastern Altai-Sayan restricts forests to isolated patches with higher soil moisture [43]–[44]. Holocene fragmentation of the alpine grasslands to isolated patches surrounded by forests could have lead to the extinction of some large mammals typical of the mammoth steppe. Sharp separation of originally intermixed faunal elements into distinct biomes seems to be the major pattern of the Last Glacial/Holocene transition [8], [45].

Most previous attempts to find a modern analogue of the mammoth steppe have been focused on regions of the Arctic tundra, i.e., Yakutia, NE Russia, and Alaska [8], [9], [46], [47]. However, present-day faunas of the Arctic regions have diverged greatly in their ecological structure from Last Glacial ones, showing more affinity to the taiga biome. The divergent ecological structure of modern Yakutian fauna is determined mainly by Holocene immigrant species, characterized by a high proportion of arboreal herbivores and predators of invertebrates associated with forest and taiga biomes (Fig. 4).

The modern arctic tundra is characterized by low productivity and relatively high homogeneity [4], [25], in contrast to the alpine biome where tundra vegetation occurs in association with forests and steppes, e.g. [48]–[49]. During the Last Glacial, tundra vegetation was confined to places with higher precipitation or lower evapotranspiration, e.g. in the mountains or at non-glaciated higher latitudes, often in the close vicinity of steppe [20].

Taiga and temperate forest species occurred in all of the examined Last Glacial assemblages; however their percentage was very low, with the exception of the Last Glacial assemblage of the Carpathian Mountains. This assemblage holds a special position as the site resembles Recent rather than any other Last Glacial community. The Carpathian Mountains of eastern Europe possibly represented a glacial refugium of forest vegetation and forest-dwelling animals, as suggested by Sommer & Nadachowski [50], Jankovská and Pokorný [21], Markova et al. [51], Willis et al. [28], and Willis & van Andel [52].

The main difference in trophic-size structure between present-day and fossil assemblages is the higher richness of large mammals and proportionally lower richness of small mammals in the latter. The high proportion of large herbivores observed in the Last Glacial as well as in some present-day communities is generally typical of areas with low tree cover [15] and could be further supported by the year-round availability of high quality food in the glacial steppe [10], [53]. Fossil assemblages are probably biased against small mammal species owing to fossil record incompleteness [54], [7], [29], [55]–[56]. However, the low proportion of small mammals cannot be considered completely artifactual because the present-day assemblages from the eastern Altai-Sayan region show very similar composition to Last Glacial communities. The low species richness of insectivores and aquatic predators in Last Glacial-like Altai-Sayan assemblages (and probably in other Last Glacial assemblages as well) could be due to the dry and cold climate associated with permafrost which strongly limits insectivores’ food sources [57, 48 58]. These results were confirmed using two different ecological classifications and using two independent statistical methods (NMDS, PCA). Moreover, our fossil datasets are based on large areas and a long time period to avoid taphonomic biases in species occurrence. Therefore, we suggest that potential bias in the Pleistocene data subset cannot significantly affect our results.

In general, Pleistocene assemblages were characterized by the occurrence of large herbivore and predator species associated with steppe, desert and alpine biomes. Mammalian paleocommunities classified according to biome type are relatively homogeneous, confirming the view of the mammoth steppe as a single Last Glacial biome [10]. In contrast to biome classification, the trophic-size structure of mammalian paleocommunities shows a degree of heterogeneity, comparable to the Recent localities. The trophic-size structure of communities is probably less influenced by historical factors [16]. Historical processes are considered to be the main factor promoting differences between communities from similar environments [15], [29].

Our results open new research possibilities for many aspects of Quaternary paleoecology. The Altai-Sayan region offers a possibility to study factors shaping the structure of so called non-analogue communities and explore vegetation and faunal changes associated with Pleistocene/Holocene transition. Research of soil nutrient availability and cycling in glacial-like localities can provide an insight into the carrying capacity of ice age ecosystems supporting numerous large herbivore species. Modeling the impact of climate changes on the glacial-like landscape may elucidate the process of biome diversification in the Holocene. Our result can be confirmed by thorough paleontological research of the as yet unexplored eastern Altai-Sayan region as well as by phylogeographical analyses of typical glacial species (e.g. steppe lemming or pika) including Altai-Sayan populations.

## Supporting Information

Figure S1 The projection scores of studied localities according to biome classification (NMDS analysis based on Bray-Curtis dissimilarity indices).
(DOCX)

Figure S2 The projection scores of studied localities according to biome classification without rare categories (PCA analysis).
(DOCX)

Figure S3 The projection scores of studied localities according to trophic-size classification (NMDS analysis based on Bray-Curtis dissimilarity indices).
(DOCX)

Figure S4 The projection scores of studied localities according to trophic-size classification without rare categories (PCA analysis).
(DOCX)

Table S1 Paleartic regions used in the analyses with associated references.
(DOC)

Table S2 Examined localities of Altai-Sayan region with associated references.
(DOCX)

Table S3 Percentages of biomes and trophic-size categories for regions and localities (dataset).
(XLS)

Table S4 Component loadings of the PCA and NMDS analyses.
(DOCX)

Notes S1 Taxonomic notes with associated references.
(DOC)
