# Supplementary material for: Ecological Structure of Recent and Last Glacial Mammalian Faunas in Northern Eurasia: The Case of Altai-Sayan Refugium
Source: PLoS One. 2014 Jan 13;9(1):e85056. doi: 10.1371/journal.pone.0085056 (PMC3890305; doi:10.1371/journal.pone.0085056)
Supplement: Figure S1 — The projection scores of studied localities according to biome classification (NMDS analysis based on Bray-Curtis dissimilarity indices). (DOCX) [file pone.0085056.s001.docx]

**Figure S1**.


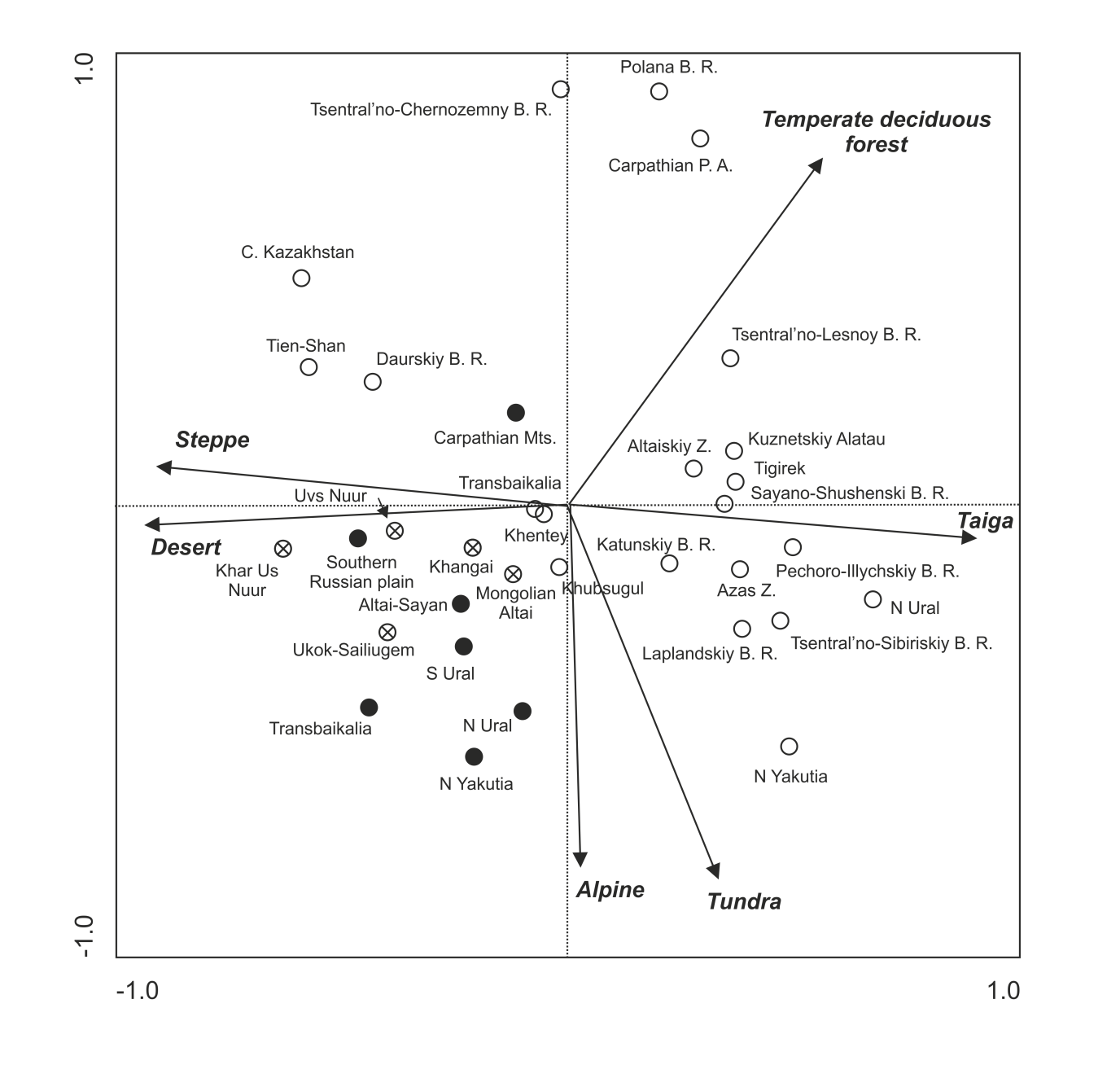


Fig. S1 The projection scores of studied localities according to biome classification (NMDS analysis based on Bray-Curtis dissimilarity indices). Open circles – Recent assemblages; crossed circles – Recent eastern Altai assemblages; full circles – Last Glacial assemblages
