# Supplementary material for: Ecological Structure of Recent and Last Glacial Mammalian Faunas in Northern Eurasia: The Case of Altai-Sayan Refugium
Source: PLoS One. 2014 Jan 13;9(1):e85056. doi: 10.1371/journal.pone.0085056 (PMC3890305; doi:10.1371/journal.pone.0085056)
Supplement: Figure S4 — The projection scores of studied localities according to trophic-size classification without rare categories (PCA analysis). (DOCX) [file pone.0085056.s004.docx]

**Figure S4**


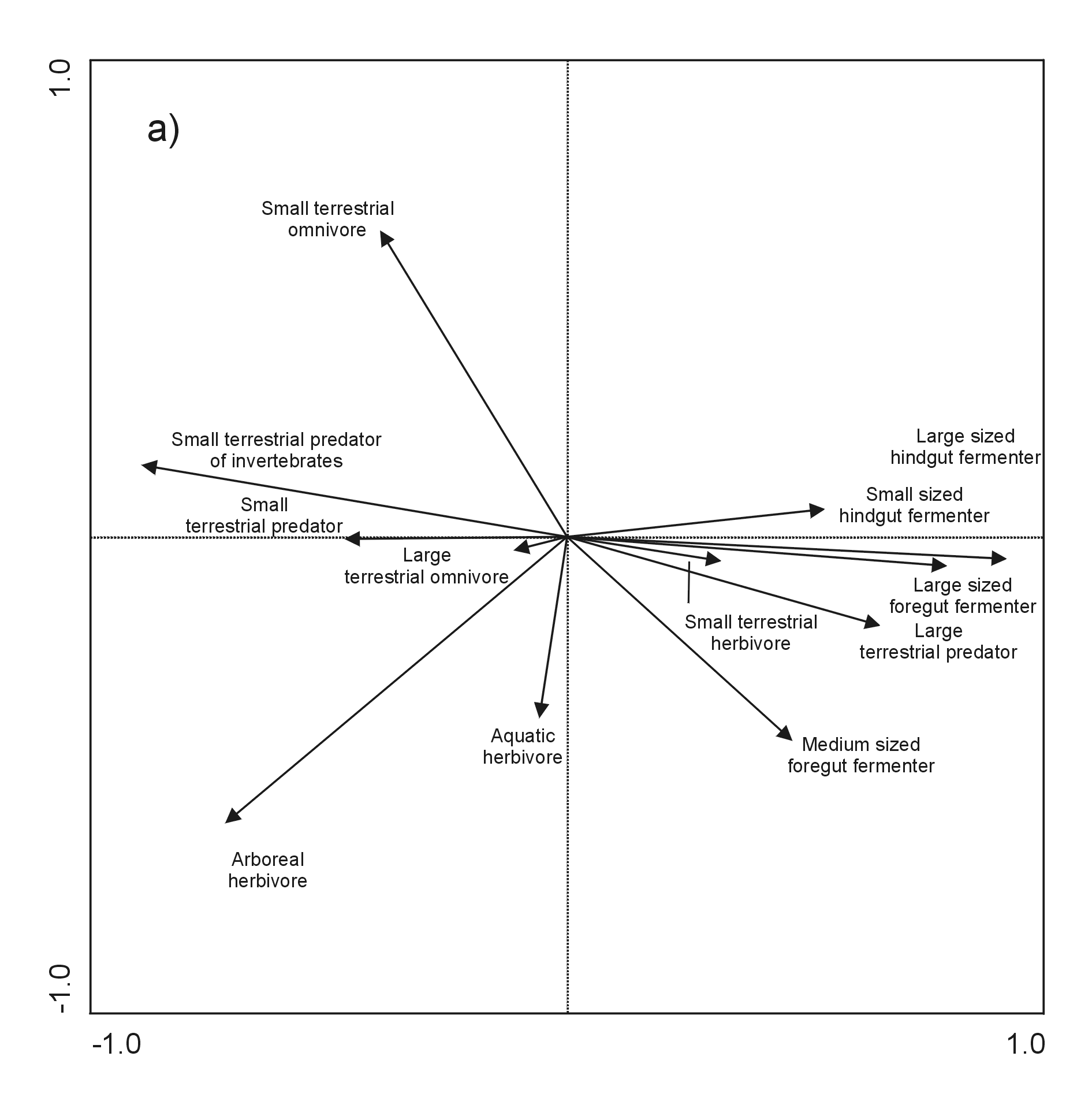


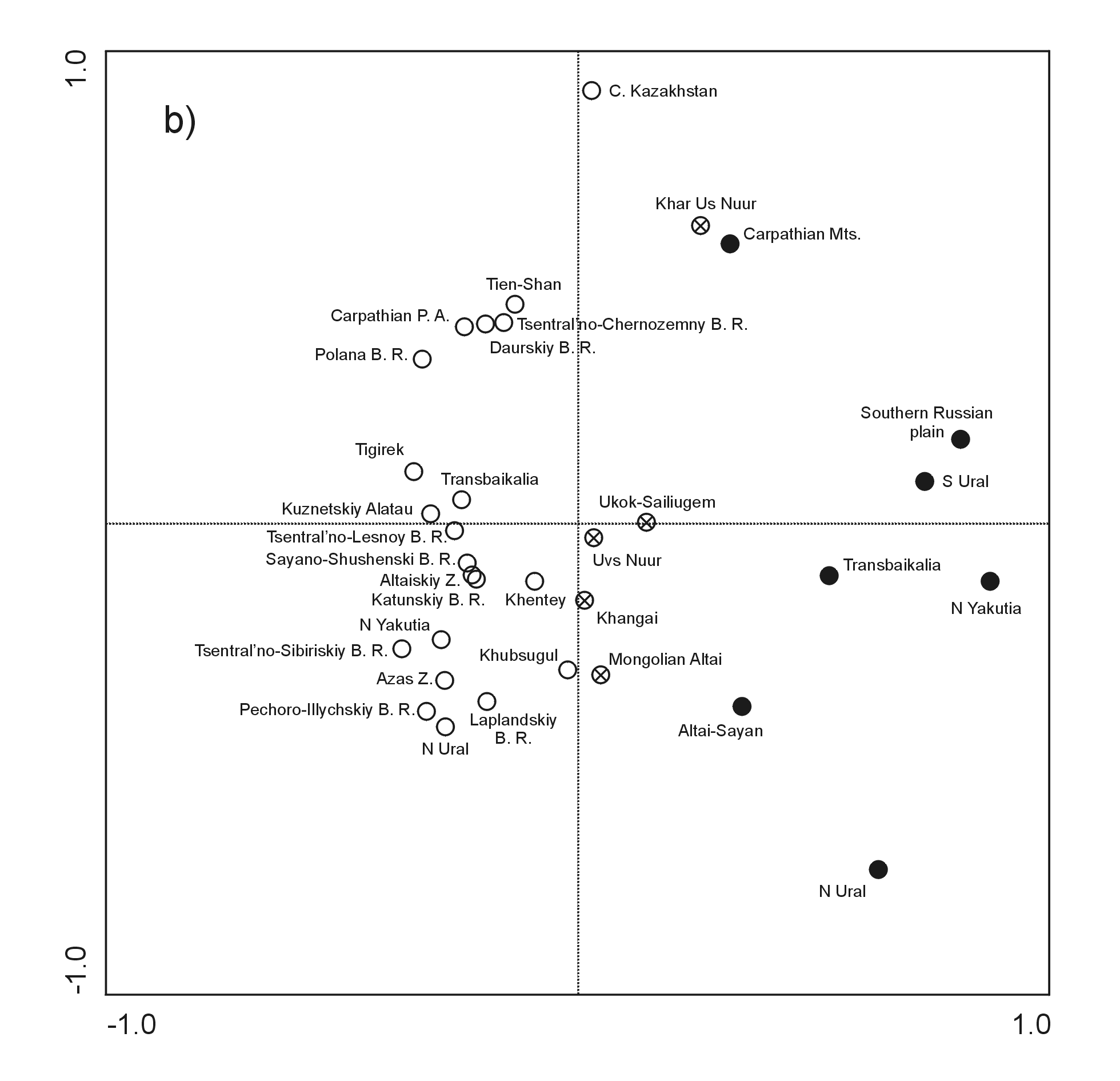


Fig. S4 The projection scores of studied localities according to trophic-sizes (PCA analysis without rare categories - aquatic predator, aquatic predator of invertebrates, subterranean predator of invertebrates, arboreal omnivore, small-sized foregut fermenter, subterranean herbivore), a) projection of trophic-sizes, b) projection of localities. Open circles – Recent assemblages; crossed circles – Recent eastern Altai assemblages; full circles – Last Glacial assemblages
