# Supplementary material for: Ecological Structure of Recent and Last Glacial Mammalian Faunas in Northern Eurasia: The Case of Altai-Sayan Refugium
Source: PLoS One. 2014 Jan 13;9(1):e85056. doi: 10.1371/journal.pone.0085056 (PMC3890305; doi:10.1371/journal.pone.0085056)
Supplement: Table S1 — Paleartic regions used in the analyses with associated references. (DOC) [file pone.0085056.s005.doc]

Table S1. Palearctic regions used in the analyses

| Region | Country | Fauna analysed |
| --- | --- | --- |
| Altai-Sayan | Russia, Mongolia, China, Kazakhstan, 48° to 54°N; 108° to 81°E | Glacial |
| Transbaikalia | Russia, Mongolia, 49° to 58°N; 105° to 120°E | Glacial & Recent |
| Northern Yakutia | Russia (limited by Lena River W, Kolyma River E, Aldan River S, incl. Chersky and Verkhovyanskiy Ridges) | Glacial & Recent |
| Southern Russian Plain | Russia and Ukraine, limited by Dnepr, Don Seversky Donets river, Volga river basin, region along Black Sea, Crimea, 17° to 27°E; 44° to 50°N | Glacial |
| North Ural | Russia, Ural Mts north of 60° N | Glacial & Recent |
| South Ural | Russia, Ural Mts south of 60° N | Glacial |
| Carpathian Mts. | The whole Carpathian Range (according to WWF ecoregion, in Czech Rep., Slovakia, Hungary, Romania, Ukraine, Serbia), 44° to 50° N; 17° to 27°E | Glacial |
| Polana Biosphere Reserve | Slovakia, Carpathian Mts., 48°39'N; 19°29'E | Recent |
| Carpathian Protected Area | Western Ukraine, Carpathian Mts., 48°03'N; 24°12'E (near city of Uzhhorod) | Recent |
| Laplandskiy Biosphere Reserve | Russia (Russian Plains region): western part of the Kola Peninsula, 67°10' to 68°05'N; 31°45' to 32°45'E | Recent |
| Tsentral’no-Lesnoy Biosphere Reserve | Russia (Russian Plains region): in the Kalinin Oblast', 56°30'N; 32°52'E | Recent |
| Tsentral’no-Chernozemny Biosphere Reserve | Russia (Russian Plains region): 51°00'N; 36°40'E | Recent |
| Pechoro-Ilychskiy Biosphere Reserve | Russia (Europe): 240 km S of city of Pechora on the western slopes of the Northern Ural Mountains. Mountain unit: 61°58' to 63°16'N; 57°47' to 59°39'E. Plain unit: 61°43' to 61°53'N; 56°52' to 57°07'E | Recent |
| Tsentral’no-Sibirskiy Biosphere Reserve | Russia (Asia): along Yenisey River, with Siberian lowland to its we st and the more elevated Siberian Plateau to its east, 60°55' to 63°15'N; 84°15' to 92°30'E | Recent |
| Khentey Mountains | North Mongolia, near Ulaanbaatar | Recent |
| Daurskiy Biosphere Reserve | Russia (Transbaikal region): 49°55' to 50°14'N; 115°05' to 115°98'E, next to the border with Mongolia, 700 km SE of Lake Baikal. | Recent |
| Central Kazakhstan | W border: Turgai region, E: Kalba and Tarbagatai Ranges, S: Turan lowland and Chu river, N: Irtysh plain. 44° to 52° N; 62° to 78° E | Recent |
| Tien-Shan Mts. | Kazakhstan, Kyrgyzstan, China: 40° to 46° N; 68° to 90° E | Recent |

Studies included in the Table 1

Agadjanian AK, Serdyuk NV (2005) The history of mammalian communities and paleogeography of the Altai Mountains in the Paleolithic. Paleontological Journal 39: 645–821.

Alexeeva NV, Erbajeva MA (2008) Diversity of Late Neogene-Pleistocene small mammals of the Baikalian region and implications for paleoenviroment and biostratigraphy: An overview. Quat Int 179: 190–195.

Andrenko OV, Ovodov ND, Zazhigin VS, Chekha VP (1999) Quaternary rodents of the NE Part of the Altai–Sayan Mountain Region. Antropozoikum 23: 117–118.

Aristov AA, Baryshnikov GF (2001) The mammals of Russia and adjacent territories. Carnivores and pinnipeds. Santkt-Peterburg: Izdateľstvo Zoologicheskogo Instituta RAN. 560 p. (In Russian).

Averianov A, (2001). Pleistocene lagomorphs of Eurasia. Deinsea 8: 1–13.

Bachura O, Kosintsev P (2007) Late Pleistocene and Holocene small- and large-mammal faunas from the Northern Urals. Quat Int 160: 121–128.

Baryshnikov GF (1999) Chronological and geographical variability of *Crocuta spelaea* (Carnivora, Hyaenidae) from the Pleistocene of Russia. Deinsea 6: 155–173.

Beketov AB, Erzhanov NT, Kapitonov VI, Slavchenko NP, Berber AP, et al. (2005) Rare and endangered animals of Central Kazakhstan hills. Pavlodar: PGU im. S. Toraigilova. (In Russian).

Bobrinskii NA, Kuznecov BA, Kuzyakin AP (1965) Guide to the mammals of the USSR. Moscow: Prosveshcheni. 382 p.

Bocherens H, Pacaud G, Lazarev PA, Mariotti A (1996) Stable isotope abundances (^13^C, ^15^N) in collagen and soft tissues from Pleistocene mammals from Yakutia: implications for the paleobiology of the mammoth steppe. Palaeogeogr Palaeoclimatol Palaeoecol 126: 31–44.

Boeskorov GG (2001) Systematics and distribution of sheep of the genus *Ovis* (Artiodactyla, Bovidae) in Eastern Siberia and the Far East in the Pleistocene and Holocene. Zool Zhurnal 80: 243–256. (In Russian).

Boeskorov GG (2005) Taxonomic Position of the Red Deer *Cervus elaphus* L. (Cervidae, Artiodactyla, Mammalia) from the Neopleistocene of Northeastern Asia (in Russian). Paleontologicheskii Zhurnal 39: 73–84. (In Russian).

Boeskorov GG (2006) Arctic Siberia: refuge of the Mammoth fauna in the Holocene. Quat Int 142–143: 119–123.

Boeskorov GG, Mol D (2004) Quaternary mammal collections in the Museum of Yakutsk (Eastern Siberia, Yakutia, Russia). Cranium 21: 19-32.

Chlachula J (2001a) Pleistocene climate change, natural enviroments and palaeolithic occupation of the Altai area, west-central Siberia. Quat Int 80–81: 131–167.

Chlachula J (2001b) Pleistocene climate change, natural enviroments and paleolithic occupation of the Angara-Baikal area, east Central Siberia. Quat Int 80–81: 69–92.

David A, Nadachowski A, Pascaru V, Wojtal P, Borziac I (2003) Late Pleistocene fauna from the Late Palaeolithic butchering site Cosăuţi 1, Moldova. Acta Zool Cracov 46: 85–96.

Erbajeva MA, Alexeeva NV (2000) Pliocene and Pleistocene biostratigraphic succession of Transbaikalia with emphasis on small mammals. Quat Int 68–71: 67–75.

Erbajeva M, Alexeeva N, Khenzykhenova F (2006) Review of the Pliocene-Pleistocene arvicolids of the Baikalian region. Palaeontographica A 278: 113–123.

Erzhanov N.T (2000) Mammals of Kazakhstan – fauna, biology and conservation. Almaty: Kazakhskii protivochumnyi nauchno-issledovatel’kii institut. (In Russian).

Fetisov AS (1956). Current zoogeographic regions of Selengi Dauria according to mammalian data. Zool Zhurnal 35: 1535–1540. (In Russian).

Finlayson C, Carrión JS (2007) Rapid ecological turnover and its impact on Neanderthal and other human populations. Trends Ecol Evol 22: 213–222.

Flint VE, Čugunov JuD, Smirin VM (1965) Mammals of USSR. Moscow: Izdatelstvo Mysl. 437 p. (In Russian).

Foronova IV (1999) Quaternary mammals and stratigraphy of the Kuznetsk Basin (South-Western Siberia). Antropozoium 23: 71–97.

Geiser F (2002) Checkliste der Säugetierarten des Altai nach Belegen des Siberian Zoological Museum in Novosibirsk: Gebiete Altai Republic und Altaiskyi Krai territory. Available: <http://www.naturatours.ch/PDF/Saeugetiere_Altai.pdf>. Accessed January 2012.

Görner M, Hackenthal H (1987) Säugetiere Europas. Leipzig-Radebeul: Neumann Verlag. 370 p.

Groves CP (1974) Horses, asses and zebras in the Wild. London: Davis and Charles. 176 p.

Heptner VG, Naumov NP, Jürgenson PB, Sludski AA, Cirkova AF, Bannikov AG (1974) Die Säugetiere der Sowjetunion, Band II: Seekühe und Raubtiere. Jena: VEB Gustav Fischer Verlag.1006 p.

Horáček I, Hanák V, Gaisler J (2000) Bats of the Palearctic region: a taxonomic and biogeographic review. Proceedings of the VIIIth EBRS 1: 11–157.

ICE Biological invertories of world protected areas. Available: <http://www.ice.ucdavis.edu/bioinventory/bioinventory.html>. Accessed January 2012.

Khenzykhenova F (1996) Late Pleistocene small mammals from the Baikal region (Russia). Acta Zool Cracov 39: 229–234.

Khenzykhenova FI (2008) Paleoenviroments of Palaeolithic humans in the Baikal region. Quat Int 179: 53–57.

Knapp M, Rohland N, Weinstock J, Baryshnikov G, Sher A, et al. (2009) First DNA sequences from Asian cave bear fossils reveal deep divergences and complex phylogeographic patterns. Molecular Ecology 18: 1225–1238.

Kosintsev PA (1996) Late Pleistocene megamammals of the Urals. Acta Zool Cracov 39: 245–250.

Kosintsev PA (2007) Late Pleistocene large mammal faunas from the Urals. Quat Int 160: 112–120.

Kurtén B (1968) Pleistocene mammals of Europe. London: Weidenfeld and Nicolson. 352 p.

Lazarev PA, Tomskaja AI (1987) Mammals and biostratigraphy of the Late Pleistocene of the northern Yakutia. Jakutsk, Akademija nauk SSSR. 169 p. (In Russian).

Lbova LV (1999) The palaeoecological model of the Upper Palaeolithic site Kamenka (Buryatia-Siberia). Antropozoikum 23: 181–191.

Lister A (2004) The impact of Quaternary Ice Ages on mammalian evolution. Philos Trans R Soc Lond B Biol Sci 359: 221–241.

Lister AM (1994) The evolution of the giant deer, *Megaloceros giganteus* (Blumenbach). Zool J Linn Soc 112: 65–100.

Litvinov NI, Bazardorzh D (1992) [Mammals of the Khubsugul region, Mongolian People's Republic](http://www.pensoft.net/notes/13291.stm). Irkutsk: Irkutsk University Press. 128 p. (In Russian).

**Markova AK (1984) Late Pleistocene Mammal Fauna of the Russian Plain. In: Velichko AA, Wright HE Jr, Barnosky CW, editors. Late Quaternary Environments of the Soviet Union. London: Longman. pp. 209–218.**

Markova AK, Smirnov NG, Kozharinov AV, Kazantseva NE, Simakova AN, et al. (1995) Late Pleistocene distribution and diversity of mammals in Northern Eurasia (PALEOFAUNA database). Paleontologia i Evolució 28–29: 5–143.

Markova A, Puzachenko A (2008a) Mammal assemblages during the Last Glacial Maximum (LGM) (≤24 - ≥ 17 kyr BP). In: Markova AK, van Kolfschoten T, Bohncke S, Kosintsev PA, Mol J, et al., editors. Evolution of European ecosystems during Pleistocene-Holocene transition (24-8 kyr BP). Moscow: KMK Scientific Press. pp. 91–116.

Markova A, Puzachenko A (2008b) Mammal assemblages during the Last Glacial transition (LGT) (≤17 - ≥ 12,4 kyr BP). In: Markova AK, van Kolfschoten T, Bohncke S, Kosintsev PA, Mol J, et al., editors. Evolution of European ecosystems during Pleistocene-Holocene transition (24-8 kyr BP). Moscow: KMK Scientific Press. pp. 117–160.

Markova AK, Puzachenko AYu, van Kolfschoten T. (2010) The North Eurasian mammal assemblages during the end of MIS 3 (Brianskian-Late Karginian-Denekamp Interstadial). Quat Int 212: 149–158.

Matveev VA, Kruskop SV, Kramenov DA (2005) Revalidation of *Myotis petax* Hollister, 1912 and its new status in connection with *M. daubentonii* (Kuhl, 1817) (Vespertilionidae, Chiroptera). Acta Chiropt 7: 23–37.

Mazák V (1981) *Panthera tigris*. Mammalian Species 152: 1–8.

Mosin V (2000) The southern Transurals in the stone age. Izvesti Celjabinskogo naucnogo scentra 2: 82–83.

Musil R (1985) Paleobiography of terrestrial communities in Europe during the Last glacial. Acta Musei Nationalis Pragae 41: 1–84.

Nadachowski A (1982) Late Quaternary rodents of Poland with special reference to morphotype dentition analysis of voles. Kraków: Polska Akademia Nauk, Kraków. 108 p.

Nikolskiy PA (2002) Late Pleistocene mammals of New Siberia Island (Russian Arctic). 32nd Annual Arctic Workshop Abstracts: 3.

Orlova LA, Kuzmin YV, Dementiev VN (2004) A review of the evidence for extinction chronologies for five species of Upper Pleistocene megafauna in Siberia. Radiocarbon 46: 301–314

Panteleyev PA (1998) The rodents of the Palaearctic, composition and areas. Moscow: Russian Academy of Sciences, Moscow. 116 p.

Pazonyi P (2004) Mammalian ecosystem dynamics in the Carpathian Basin during the last 27 000 years. Palaeogeogr Palaeoclimatol Palaeoecol 212: 295–314.

Rekovets LI (1995) Periglacial micromammal faunas from the Late Pleistocene of Ukraine. Acta Zool Cracov 38(1): 129–138.

Rieger I (1981) *Hyaena hyaena*. Mammalian Species 150: 1–5.

Rossina VV (2006) Bats as an indicator of human activity in the Paleolithic, using the example of Denisova Cave, northwestern Altai. Paleontological Journal 40: 494–500.

Rzebik-Kowalska B (2007) New data on Soricimorpha (Lipotyphla, Mammalia) from the Pliocene and Pleistocene of Transbaikalia and Irkutsk Region (Russia). Acta Zool Cracov 50: 15–48.

Sher AW (1971) Säugetierfunde und Pleistozänstratigraphie in der Kolyma-Niederung. Berichte der deutschen Gesellschaft für geologische Wissenschaften A 16: 113–125.

Smith FH, Janković I, Karavanić I (2005) The assimilation model, modern human origins in Europe, and the extinction of Neanderthals. Quat Int 137: 7–19.

Sokolov II (1959) Fauna of USSR, Mammals, Ungulates (Perissodactyla and Artiodactyla. Moscow: Izdatelstvo Akademii Nauk SSSR. 639 p.

Sokolov VE (1974) *Saiga tatarica*. Mammalian Species 38: 1–4.

Sommer R, Benecke N (2004) Late- and Post-glacial history of the Mustelidae in Europe. Mamm Rev 34: 249–284.

Sommer R, Benecke N (2006) Late Pleistocene and Holocene development of the felid fauna (Felidae) of Europe: a review. J Zool 269: 7–19.

Sommer RS, Nadachowski A (2006) Glacial refugia of mammals in Europe: evidence from fossil record. Mamm Rev 36: 251–265.

Stuart AJ, Kosintsev PA, Higham TFG, Lister AM (2004) Pleistocene to Holocene extinction dynamics in giant deer and wooly mammoth. Nature 431: 684–689.

Sunquist M, Sunquist F (2002) Wild cats of the World. Chicago: University of Chicago Press. 452 p.

Titov VV (2008) Habitat conditions for *Camelus knoblochi* and factors in its extinction. Quat Int 179: 120–125.

Vereshchagin NK (1959) Mammals of Caucasus (history of faunal development). Moscow: Izdatelstvo Akademii Nauk SSSR. 703 p. (In Russian).

Wilson DE, Reeder D-AM (2005) Mammal species of the World. A taxonomic and geographic reference. Baltimore: Johns Hopkins University Press. 2142 p.

Yudin BS, Galkina LI, Potapkina AF (1979*)* Mammals of the Altai-Sayan mountainous part. Novosibirsk: Nauka. 296 p. (In Russian).

Zagorodnyuk I (2002) Field key to small mammals of Ukraine (in Russian). Proceedings of the Theriological School 5: 1–60.

Zazula GD, Froese DG, Elias SA, Kuzmina S, Mathewes RW (2007) Arctic ground squirrels of the mammoth-steppe. Paleoecology of Late Pleistocene middens (~24000-29 450 ^14^C yr BP), Yukon Territory, Canada. Quat Sci Rev 26: 979–1003.
