# Supplementary material for: Ecological Structure of Recent and Last Glacial Mammalian Faunas in Northern Eurasia: The Case of Altai-Sayan Refugium
Source: PLoS One. 2014 Jan 13;9(1):e85056. doi: 10.1371/journal.pone.0085056 (PMC3890305; doi:10.1371/journal.pone.0085056)
Supplement: Table S2 — Examined localities of Altai-Sayan region with associated references. (DOCX) [file pone.0085056.s006.docx]

Table S2. Examined localities of Altai-Sayan region.

| Locality | Biome | Climate | Coordinates |
| --- | --- | --- | --- |
| Tigireksky Zapovednik | T, DF, G, AG | temp. 0.8˚C, -13.8 to 15.2˚C  prec. 516 to 1500 mm  snow 600 to 2000 mm | N 50°58’47’’  E 82°54’25’’ |
| Kuznetsky Alatau | T, DF, AG | temp. -15.5 to 16.5˚C  prec. 566 mm  snow max. 4000 mm | N 54°34’18’’  E 88°12’2’’ |
| Sayano-Shushensky Biosphere Reserve | T, DF, AG | temp. -3˚C, -20 to 20˚C  prec. 750 mm  snow 300 to 1500 mm | N 52° 8’ 51’’  E 92° 4’ 34’’ |
| Azas Zapovednik | T, DF, G, AG | temp. -5.5˚C, -29 to 15˚C  prec. 343 to 800 mm  snow 300 to 800 mm | N 52°31’37’’  E 97°28'53"' |
| Altaisky Zapovednik | T, DF, GA, G | temp. -19.7 to 16˚C  prec. 250-900 mm  snow 1500 mm | N 51°1’56’’  E 88°39’31’’ |
| Katunskiy Zapovednik | T, DF, G, AG | temp. -13.2 to 5.7˚C  prec. 882 mm  snow 1000 mm | N 49°41’31’’  E 86°5’10’’ |
| Khubsugul | T, G, AG | temp. -4.5˚C, -26.5 to 14˚C  prec. 247 mm  snow 150 mm | N 51°11’5’’  E 100°35’26’’ |
| Ukok-Sailiugem | G, AG, D | temp. -8.3˚C, -28 to 9˚C  prec. 200 mm,  snow 150 mm | N 49°15’  E 87°30’ |
| Uvs Nuur National Park | T, G, AG, D | temp. -3.5˚C, -33 to 21˚C  prec. 200 mm,  snow 100 mm | N 49°46’–50°40’  E 90°12’–95°38’ |
| Khar Us Nuur National Park | G, D | temp. -2˚C, -24 to 20˚C  prec. 100 mm  snow <100 mm | N 47°57’44’’  E 92°41’52’’ |
| Mongolian Altai | T, DF, G, AG, D | temp. -0.3˚C, -25.3 to 18˚C  prec. 134 mm  snow 100 mm | N 48°34’42’’  E 88°33’23’’ |
| Khangai | T, G, AG | temp. -6.2˚C, -32 to 15.4˚C  prec. 194 mm  snow <200 mm | N 47°42’47’’  E 99°28’34’’ |

Abbreviations and explanations: Biome categories – T = taiga, DF = deciduous forest, G = grassland, AG = alpine grassland, D = desert; Climate categories – temp. = temperature (average when available, and range), prec. = precipitation in mm, snow = snowfall in mm.

Studies included in the Table 2

Bannikov AG (1954) The mammals of the Mongolian People's Republic. Moscow: Akademiya Nauk SSSR. 669 p. (In Russian).

**Biodat database. Available:** <http://www.biodat.ru/db/vid/index.htm>**.** Accessed January 2012**.**

[Grunert J](http://apps.isiknowledge.com/WoS/CIW.cgi?SID=T12jNil8cOhndanmblF&Func=OneClickSearch&field=AU&val=Grunert+J&curr_doc=2/3&Form=FullRecordPage&doc=2/3), [Lehmkuhl F](http://apps.isiknowledge.com/WoS/CIW.cgi?SID=T12jNil8cOhndanmblF&Func=OneClickSearch&field=AU&val=Lehmkuhl+F&curr_doc=2/3&Form=FullRecordPage&doc=2/3), [Walther M](http://apps.isiknowledge.com/WoS/CIW.cgi?SID=T12jNil8cOhndanmblF&Func=OneClickSearch&field=AU&val=Walther+M&curr_doc=2/3&Form=FullRecordPage&doc=2/3) (2000) Paleoclimatic evolution of the Uvs Nuur basin and adjacent areas (Western Mongolia) Quat Int 65–66: 171–192.

Hilbig W (2003) The distribution of the vegetation in the Uvsnuur basin and its surrounding mountain ranges. Feddes Repert 114: 540–558.

Hoare R (2003) WorldClimate Database. Available: http://www.worldclimate.com. Accessed January 2012**.**

Kharlamova NF (2004) Climatic characteristics of Ukok Plateau and adjoining territories. The News of Altai State University Barnaul 3: 71–77.

Litvinov NI, Bazardorzh D (1992) [Mammals of the Khubsugul region, Mongolian People's Republic](http://www.pensoft.net/notes/13291.stm). Irkutsk: Irkutsk Univ Press, Irkutsk). 128 p. (In Russian).

[Luo SJ](http://apps.isiknowledge.com/WoS/CIW.cgi?SID=V2I1hMo9ALHjheLdf@j&Func=OneClickSearch&field=AU&val=Luo+SJ&curr_doc=1/1&Form=FullRecordPage&doc=1/1), [Kim JH](http://apps.isiknowledge.com/WoS/CIW.cgi?SID=V2I1hMo9ALHjheLdf@j&Func=OneClickSearch&field=AU&val=Kim+JH&curr_doc=1/1&Form=FullRecordPage&doc=1/1), [Johnson WE](http://apps.isiknowledge.com/WoS/CIW.cgi?SID=V2I1hMo9ALHjheLdf@j&Func=OneClickSearch&field=AU&val=Johnson+WE&curr_doc=1/1&Form=FullRecordPage&doc=1/1), [van der Welt J](http://apps.isiknowledge.com/WoS/CIW.cgi?SID=V2I1hMo9ALHjheLdf@j&Func=OneClickSearch&field=AU&val=van+der+Welt+J&curr_doc=1/1&Form=FullRecordPage&doc=1/1), [Martenson J](http://apps.isiknowledge.com/WoS/CIW.cgi?SID=V2I1hMo9ALHjheLdf@j&Func=OneClickSearch&field=AU&val=Martenson+J&curr_doc=1/1&Form=FullRecordPage&doc=1/1),, et al. (2004) Phylogeography and genetic ancestry of tigers (*Panthera tigris*). PLoS Biol 2: 2275–2293.

Malkov NP (1996) Red Data Book of the Altai Republic. Novosibirsk: RIO Univer-Print GAGU. 256 p. (In Russian).

Mallon D (2005) *Saiga tatarica* ssp. *mongolica*. IUCN Red List of Threatened Species. Available: [www.iucnredlist.org](http://www.iucnredlist.org/). Accessed January 2012.

Marinin AM, Malkov NP, Goverdovskii VA, Coenov VI, Maneev AG, vet al. (2002) The Red Book of the Republic Altai: Specially protected territories and objects. Gorno Altaisk: WWF. 272 p. (In Russian).

McGinley M (2007) Great Lakes Basin desert steppe, Encyclopedia of Earth. Available: <http://www.eoearth.org/article/Great_Lakes_Basin_desert_steppe>. Accessed January 2012.

OOPT RF database (Protected areas of Russian Federation). Available: <http://zapoved.ru/>. Accessed January 2012.

# Sokolov VE, Orlov VN (1980) Field Guide to Mammals of Mongolia. Moscow: Nauka. 351 p. (In Russian).

Tao D (2002) The fossil of the Przewalski´s horse and the climatic variations of the Late Pleistocene in China. In: Marhkour M, editor. Equids in time and space. Oxford: Oxbow Books. pp. 12–19.

Tupikova NV (1989). Area structure of rodents and lagomorphs of the Altai. Fauna and Ecology of Rodents 17: 59–114.

World Database on Protected Areas. Available: <http://www.unepwcmc.org/wdpa/index.htm>. Accessed January 2012.

Yudin BS, Galkina LI, Potapkina AF (1979*)* Mammals of the Altai-Sayan mountainous part. Novosibirsk: Nauka. 296 p. (In Russian).
