# Supplementary material for: Ecological Structure of Recent and Last Glacial Mammalian Faunas in Northern Eurasia: The Case of Altai-Sayan Refugium
Source: PLoS One. 2014 Jan 13;9(1):e85056. doi: 10.1371/journal.pone.0085056 (PMC3890305; doi:10.1371/journal.pone.0085056)
Supplement: Table S4 — Component loadings of the PCA and NMDS analyses. (DOCX) [file pone.0085056.s008.docx]

Table S4. Component loadings of the PCA and NMDS analyses.

| Dataset | Analysis | Value | Axis 1 | Axis 2 | Axis 3 | Axis 4 |
| --- | --- | --- | --- | --- | --- | --- |
| Biomes | NMDS | Cumulative explained variation | 65.31 | 94.15 | 100.00 |  |
| Biomes | PCA | Cumulative explained variation | 65.70 | 89.90 | 95.22 | 98.570 |
| Biomes | NMDS | Eigenvalue | 0.6531 | 0.2884 | 0.0585 |  |
| Biomes | PCA | Eigenvalue | 0.6570 | 0.2420 | 0.0532 | 0.034 |
| Trophic-size | NMDS | Cumulative explained variation | 64.50 | 83.36 | 100.00 |  |
| Trophic-size | PCA | Cumulative explained variation | 47.68 | 63.43 | 75.18 | 80.150 |
| Trophic-size | NMDS | Eigenvalue | 0.6450 | 0.1887 | 0.1664 |  |
| Trophic-size | PCA | Eigenvalue | 0.4768 | 0.1576 | 0.1175 | 0.050 |
| Biomes (without rare categories) | PCA | Cumulative explained variation | 59.37 | 96.32 | 99.52 | 100.00 |
| Biomes (without rare categories) | PCA | Eigenvalue | 0.5937 | 0.3695 | 0.0320 | 0.0048 |
| Trophic-size (without rare categories) | PCA | Cumulative explained variation | 58.82 | 70.06 | 79.93 | 85.72 |
| Trophic-size (without rare categories) | PCA | Eigenvalue | 0.5882 | 0.1125 | 0.0986 | 0.0579 |
