# Supplementary material for: Ecological Structure of Recent and Last Glacial Mammalian Faunas in Northern Eurasia: The Case of Altai-Sayan Refugium
Source: PLoS One. 2014 Jan 13;9(1):e85056. doi: 10.1371/journal.pone.0085056 (PMC3890305; doi:10.1371/journal.pone.0085056)
Supplement: Notes S1 — Taxonomic notes with associated references. (DOC) [file pone.0085056.s009.doc]

**Notes S1: Species list, taxonomy, and distribution**

For taxa names and Recent distribution, we followed Wilson & Reeder [1], except for horses, cattle, goats, and sheep where we prefer to use specific names for the wild ancestors and domesticated forms [2]. The globally extinct species are marked by “†”. No subspecific ranks were used. As the fossil record is often very fragmentary, we sometimes applied only the supraspecific ranks (e.g. “*Ochotona* sp.”) if the reliable species determination was not possible.

The Last Glacial species distribution was derived exclusively from the fossil record (possible implications of the results of molecular phylogeography were not assessed).

**Autochtonous and allochtonous elements**

Recent mammalian communities are referred to the interval from now to approx. 16^th^ century AD. They include autochtonous elements extinct in the historical time, but not the allochtonous elements associated with the human unintended or intentional activities. Allochtonous elements are often easily detectable on the basis of historical faunal succession (e. g. *Macropus rufogriseus*, *Nyctereutes procyonoides*, *Procyon lotor*, *Neovison vison*, *Muntiacus reevesi*, *Axis axis*, *Cervus nippon*, *Odocoileus virginianus*, *Hydropotes inermis*, *Ammotragus lervia, Castor canadensis*, *Ondatra zibethicus*, *Myocastor coypus* – all in Europe; see [3]) or phylogeographic evidence (e.g. *Macaca sylvanus* in Europe; see [4]); however, some species remain problematic (e.g. autochtonous European distribution of *Atelerix algirus*, *Genetta genetta*, *Herpestes ichneumon*; see [3, 5]. In general, we tend to consider the problematic forms allochtonous with a few exceptions (see Supporting Table below). Some species could be autochtonous in some regions, but not in the others (*Oryctolagus cuniculus* autochtonous in the Iberian Peninsula only) – see Table 1 below. The marine mammals (cetaceans, pinnipeds) and island endemics (although geographically associated with the analysed regions, e.g. †*Nesiotites* spp., † *Hypnomys* spp. and †*Myotragus balearicus* in Balearic Is.; †*Prolagus sardus*, †*Tyrrhenicola henseli* and †*Rhagamys orhodon* in Corsica and Sardinia; *Plecotus sardus* in Sardinia, †*Leithia melitensis* in Corsica and Malta; *Crocidura sicula* in Sicily, Egadi Is., Ustica, and Gozo; *C. zimmermanni* and *Acomys minous* in Crete; *Dicrostonyx vinogradovi* and *Lemmus portenkoi* in Wrangel I. – for detail see [1, 6]) were excluded from analyses. Domesticated mammals were not considered in our analyses.

Supporting Table. Autochtonous or allochtonous status of “problematic” recent species in studied areas.

| Species | Status and region | References |
| --- | --- | --- |
| ***Macaca sylvanus*** | introduced into Iberian Peninsula | 1, 4 |
| ***Oryctolagus cuniculus*** | autochtonous in Iberian Peninsula and southern France only; introduced worlwide | 1 |
| *Atelerix algirus* | introduced into Iberian Peninsula and southern France | 1, 5 |
| *Genetta genetta* | introduced into Iberian Peninsula and southern France | 5 |
| *Herpestes auropunctatus* | introduced into Balkan Peninsula | 3 |
| *Herpestes ichneumon* | introduced into Iberian Peninsula | 5 |
| *Nyctereutes procyonoides* | introductions into Europe | 1 |
| *Neovison vison* | introductions into Palearctic region | 1 |
| *Procyon lotor* | introductions into Palearctic region | 1 |
| *Odocoileus virginianus* | introductions into Europe | 1 |
| *Axis axis* | introduced into Balkan Peninsula | 3 |
| *Cervus nippon* | introductions into Europe | 1 |
| ***Dama dama*** | autochtonous in southern Turkey only; introductions into nearly all countries of Europe | 1 |
| *Ammotragus lervia* | introduced into Iberian Peninsula and central Europe | 3 |
| *Sciurus carolinensis* | introduced into Italy | 1 |
| *Callosciurus erythraeus* | introduced into southern France | 3 |
| *Callosciurus finlaysonii* | introduced into Italy | 3 |
| ***Tamias sibiricus*** | introduced into western, central and southern Europe (e.g. Germany, Italy) | 3 |
| *Castor canadensis* | introductions into Palearctic region | 1, 3 |
| *Ondatra zibethicus* | introductions into Palearctic region | 1 |
| *Mus spretus* | introduced into Iberian Peninsula and southern France | 1, 5 |
| *Rattus norvegicus* | autochtonous in southeastern Siberia, northern China and Hondo region of Japan only; introduced worldwide | 1 |
| *Rattus rattus* | autochtonous in Indian Peninsula only; introduced worldwide | 1 |
| ***Hystrix cristata*** | autochtonous in Europe based on fossil record and morphology | 7 |
| *Myocastor coypus* | introductions into Palearctic region | 1 |

Introductions outside studied areas (e.g. *Macropus rufogriseus , Muntiacus reevesi, Hydropotes inermis* in British Isles) are not mentioned. Species present in our dataset, due to their recent autochtonous status for particular areas or former distribution in Pleistocene in studied areas, are in bold.

**Fused taxa**

As neontologists have more available information (soft morphology, karyology, molecular biology, behavior, ecology) than paleontologists, they are able to determine mammalian taxa more precisely. Some recent species are not diagnosable in the fossil record owing to absence of the diagnostic morphological, cytogenetic, or molecular characters, and/or fragmentation of fossil remains. Some fossil (and sometimes even recent) species are in fact paraphyletic assemblages of populations that are not specifically related. We therefore fused some closely related mammalian species into single operational taxonomic units – for detailed arguments see [1, 6, 8–20].

List of the fused mammalian species (the order follows [1])

- *Lepus capensis* s. l. = *L. capensis* s. str. *+ L. castroviejoi + + L. corsicanus + L. europaeus + L. granatensis + L. tibetanus + L. tolai*
- *Erinaceus europaeus* s. l. = *E. concolor* + *E. europaeus* s. str. + *E. roumanicus*
- *Crocidura suaveolens* s. l. = *C. gueldenstaedtii* + *C. russula* + *C. suaveolens* s. str.
- *Sorex araneus* s. l. = *S. antinorii + S. araneus* s. str. *+ S. arunchi + S. coronatus + S. samniticus*
- *Sorex tundrensis* s. l. = †*S. runtonensis* (syn. *sibiriensis, arcticus) + S. tundrensis* s. str.
- *Eptesicus nilssonii s. l. = E. bobrinskoi* + *E. nilssonii* s. str.
- *Pipistrellus pipistrellus* s. l. = *P. pipistrellus* s. str. + *P. pygmaeus*
- *Barbastella barbastellus* s. l. *= B. barbastellus* s. str. *+ B. leucomelas*
- *Plecotus auritus* s. l. = *P. auritus* s. str. *+ P. austriacus + P. kolombatovici + P. macrobullaris + Plecotus ognevi*
- *Myotis daubentonii* s. l. = *M. daubentonii* s. str. + *M.* *petax*
- *Myotis myotis* s. l. *= M. blythii* s. str. *+ M. myotis* s. str. *+ M. oxygnathus*
- *Myotis mystacinus* s. l. *= M. alcathoe + M. aurascens + M. hajastanicus + M. mystacinus* s. str. *+ M. nipalensis*
- *Felis silvestis* s. l. = *F. bieti* + *F. libyca + F. silvestris* s. str. + *F. ornata*
- *Panthera leo* s. l. = *P. leo* s. str. + †*P. spelaea*
- †*Canis mosbachensis* s. l. = several forms of Pleistocene canids (e.g. †*C. spelaeus*, †*C. volgensis* etc.) with highly inconsistent taxonomy
- *Meles meles* s. l. = *M. leucurus*.+ *M. meles* s. str.
- *Equus ferus* s. l. = several forms of Pleistocene and Holocene horses (e.g. †*E. ferus cracoviensis*, †*E. f. ferus*, †*E. f. latipes*, †*E. f. lenensis*, †*E. f. longipes*, *E. f. przewalskii*, †*E. f. silvestris*, †*E. f. strictipes*, †*E. uralensis*, †*E. germanicus*, †*E. mosbachensis*, †*E. woldrichi*, etc.) with highly inconsistent taxonomy
- *Hippopotamus amphibius* s. l. = *H. amphibius* s. str. + †*H. antiquus*
- *Alces alces* s. l. = *A. alces* s. str. + *A. americanus*
- *Cervus elaphus* s. l. *= C. canadensis + C. elaphus* s. str.
- *Saiga tatarica* s. l. = *S.* *borealis + S. tatarica* s. str.
- *Bos grunniens* s. l. *=* † *B. baikalensis + B. grunniens* s. str.
- *Ovibos moschatus* s. l. = *Ovibos moschatus* s. str. + †*O.* *pallantis*
- *Sicista betulina* s. l. *= Sicista betulina* s. str. *+ S. napaea + S. pseudonapaea + S. strandi*
- *Sicista subtilis* s. l. *= S. severtzovi + S. subtilis* s. str.
- *Spalax leucodon* s. l. *= S. leucodon* s. str. *+ S. nehringi*
- *Arvicola amphibius* s. l. *= A. amphibius* s. str. *+ A. sherman + A. terrestris*
- *Dicrostonyx torquatus* s. l. *=* † *D. guilelmi +* † *D. henseli + D. torquatus* s. str.
- *Microtus cabrerae* s. l. = †*M. brecciensis* + *M. cabrerae* s. str.
- *Lagurus lagurus* s. l. *= L. lagurus* s. str. *+* † *L. transiens*
- *Microtus arvalis* s. l. *= M. arvalis* s. str. *+ M. obscurus*
- *Microtus savii* s. l. *= M. brachycercus + M. savii* s. str.
- *Mus musculus* s. l. *= M. m. bactrianus + M. m. castaneus + M. m. domesticus + M. m. gentilulus* *+ M. m. musculus* s. str.

**References**

1. Wilson DE, Reeder D-AM (2005) Mammal species of the World. A taxonomic and geographic reference. Baltimore: Johns Hopkins University Press. 2142 p.

2. Gentry A, Clutton-Brock J, Groves CP (2004) The naming of wild animal species and their domestic derivatives. J Archaeol Sci 31: 645–651.

3. Mitchell-Jones AJ, Amori G, Bogdanowicz W, Kryštufek B, Reijnders PJH, et al. (1999) The atlas of European mammals. London: Academic Press. 250 p.

4. Modolo L, Salzburger W, Martin RD (2005) Phylogeography of Barbary macaques (*Macaca sylvanus*) and the origin of the Gibraltar colony. Proc Natl Acad Sci USA 102: 7392–7397.

5. Dobson M (1998) Mammal distribution in the western Mediterranean: the role of human intervention. Mamm Rev 28: 77–88.

6. Kurtén B (1968) Pleistocene mammals of Europe. London: Weidenfeld and Nicolson. 352 p.

7. Angelici FM, Capizzi D, Luiselli L (2003) Morphometric variation in the skulls of the crested porcupine *Hystric cristata* from mainland Italy, Sicily and northern Africa. Mamm Biol 68: 165–173.

8. Alberdi MT, Caloi L, Dubrovo I, Palombo MR, Tsoukala E (1998) Large mammal faunal complexes and palaeoenviromental changes in the late Middle and Late Pleistocene: a preliminary comparison between the Eastern European and the Mediterranean areas. Geologija 25: 8–19.

9. Boeskorov GG (2006) Arctic Siberia: refuge of the Mammoth fauna in the Holocene. Quat Int 142–143: 119–123.

10. Burger J, Rosendahl W, Loreille O, Hemmer H, Eriksson T, et al. (2004) Molecular phylogeny of the extinct cave lion *Panthera leo spelaea*. Mol Phylogenet Evol 30: 841–849.

11. Driscoll CA, Menotti-Raymond M, Roca AL, Hupe K, Johnson WE, et al. (2007) The Near Eastern origin of cat domestication. Science 317: 519–523.

**12. Forsten A (1988) The small caballoid horse of the upper Pleistocene and Holocene.** J Anim Breed Genet **105**: **161–176.**

13. Gromov IM, Polyakov IY (1992) Fauna of the USSR, Mammals, Vol. III, No. 8 – Voles (Microtinae). Washington D.C.: Smithsonian Institution Libraries & National Science Foundation. 725 p.

14. Groves CP (1974) Horses, asses and zebras in the Wild. London: Davis and Charles. 176 p.

15. Groves, C.P. (2007) Family Cervidae. In: Prothero DR, Foss SE, editors. The Evolution of Artiodactyls. Baltimore: Johns Hopkins University Press. pp. 249–256.

16. Horáček I, Hanák V, Gaisler J (2000) Bats of the Palearctic region: a taxonomic and biogeographic review. Proceedings of the VIIIth EBRS 1: 11–157.

17. Lister A (2004) The impact of Quaternary Ice Ages on mammalian evolution. Philos Trans R Soc Lond B Biol Sci 359: 221–241.

18. Matveev VA, Kruskop SV, Kramenov DA (2005) Revalidation of *Myotis petax* Hollister, 1912 and its new status in connection with *M. daubentonii* (Kuhl, 1817) (Vespertilionidae, Chiroptera). Acta Chiropt 7: 23–37.

19. Sunquist M, Sunquist F (2002) Wild cats of the World. Chicago: University of Chicago Press. 452 p.

20. Yamaguchi N, Cooper A, Werdelin L, Macdonald DW (2004) Evolution of the mane and group-living in the lion (*Panthera leo*): a review. J Zool 263: 329–342
